# The impact of clinical pharmacist-physician communication on reducing drug-related problems: a mixed study design in a tertiary teaching Hospital in Xinjiang, China

**DOI:** 10.1186/s12913-022-08505-1

**Published:** 2022-09-14

**Authors:** Feiyang Zheng, Dan Wang, Xinping Zhang

**Affiliations:** 1 School of Medicine and Health Management, Tongji Medical College of Huazhong University of Science and Technology, Wuhan, 430030 Hubei China; 2 School of Management, Hubei University of Chinese Medicine, Huangjiahu West Road No.16, Hongshan District, Wuhan, China

**Keywords:** Clinical pharmacists-physicians, Drug related problem, Communication mode, RCT, Polymedicine patients

## Abstract

**Background:**

The incidence of drug-related problems (DRPs) has caused serious health hazards and economic burdens among polymedicine patients. Effective communication between clinical pharmacists and physicians has a significant impact on reducing DRPs, but the evidence is poor. This study aimed to explore the impact of communication between clinical pharmacists and physicians on reducing DRPs.

**Methods:**

A semistructured interview was conducted to explore the communication mode between clinical pharmacists and physicians based on the interprofessional approach of the shared decision-making model and relational coordination theory. A randomized controlled trial (RCT) was used to explore the effects of communication intervention on reducing DRPs. Logistic regression analysis was used to identify the influencing factors of communication.

**Results:**

The mode of communication is driven by clinical pharmacists between clinical pharmacists and physicians and selectively based on different DRP types. Normally, the communication contents only cover two (33.8%) types of DRP contents or fewer (35.1%). The communication time averaged 5.8 minutes. The communication way is predominantly face-to-face (91.3%), but telephone or other online means (such as WeChat) may be preferred for urgent tasks or long physical distances. Among the 367 participants, 44 patients had DRPs. The RCT results indicated a significant difference in DRP incidence between the control group and the intervention group after the communication intervention (*p* = 0.02), and the incidence of DRPs in the intervention group was significantly reduced (15.6% vs. 0.07%). Regression analysis showed that communication time had a negative impact on DRP incidence (OR = 13.22, *p* < 0.001).

**Conclusion:**

The communication mode based on the interprofessional approach of the shared decision-making between clinical pharmacists and physicians in medication decision-making could significantly reduce the incidence of DRPs, and the length of communication time is a significant factor. The longer the communication time is, the fewer DRPs that occur.

**Trial registration:**

This trial was approved by the ethics committee of The First Affiliated Hospital of Medical College of Xinjiang Shihezi University Hospital (kj2020–087-03) and registered in the China clinical trial registry (https://www.chictr.org.cn, number ChiCTR2000035321 date: 08/08/2020).

**Supplementary Information:**

The online version contains supplementary material available at 10.1186/s12913-022-08505-1.

## Introduction

Drug-related problems (DRPs) are defined as any undesirable events experienced by a patient that are suspected to be related to drug therapy and can potentially or actually interfere with the desired health outcome [1]. DRPs due to medication errors are common, including medication discrepancies between recorded treatment plans across different medical locations [2]. Unintentional medication discrepancies may be a potential risk of medication errors that pose a significant threat to patient health and even endanger their lives [3, 4]. According to the World Health Organization (WHO), medication errors are a leading cause of avoidable harm within health care, and organizational adverse events occur in approximately one in every ten hospitalizations [5]. As the aging trend intensifies, older patients with chronic diseases are challenged by the complexities of polymedicine. Polypharmacy is reported to be a heightened risk factor for DRP occurrence [6]. Midlov’s study of elderly patients on multiple medications showed, on average, two medication errors in every care transition [7]. Recent studies have shown that over one-third of patients (35.9%) experience medical advice errors. Because of incomplete data sources and inadequate communication, 85% of patients’ errors are due to medication history (e.g., not including aspirin on the preadmission medication list), as almost half are omitted [8]. Sixty-seven percent of hospitalized patients had at least one error in their prescription drug history at the time of admission [9], and 20–87% of patients had medication discrepancies at the time of discharge [10]. Unresolved drug differences may lead to a significant increase in harmful DRPs [11].

The solution to DRPs is closely related to the communication between clinical pharmacists and physicians. Intentional communication and collaboration between clinical pharmacists and physicians can support patients with complex medication decisions and promote better health outcomes [12, 13]. Gerardo’s research showed that 90% of physicians agreed that pharmacists’ recommendations are clinically helpful, and pharmacists have increased their knowledge of the medications they prescribe. Physicians have emphasized the value of clinical pharmacist communication, team care, and medication management [14]. A qualitative survey in Ireland found that effective communication and interprofessional trust are essential for successful collaboration between pharmacists and other health professionals [15]. Lucian et al. proposed that intentional interactive communication between clinical pharmacists and physicians helps lower the rate of adverse drug events caused by prescribing errors. Medication reconciliation (MR) is a pharmaceutical service dedicated to reducing DRPs. In this process, clinical pharmacists need to inform physicians of the types of DRPs and possible adverse results and understand the basis of prescribing this drug from physicians. Effective communication can influence a consensus on medication decision-making. However, there is no standardized and effective communication plan between pharmacists and physicians on DRPs, and the communication effect lacks evidentiary support [10, 16–19].

However, many DRPs are often the result of inadequate communication across health care providers in various departments [20]. Due to the lack of clinical information about patients, the independent and parallel working systems of medical staff, and the imbalance of authority or professional boundary friction when delivering patient care, clinical pharmacists often lack effective communication with physicians [21]. Communication methods are mainly non face-to-face (such as by fax or telephone), and medical communication is mostly incomplete and fragmented [22]. In the MR process, clinical pharmacists usually report only a medication discrepancy list to the physician without further detailed discussions. Case noted studies in the USA found that DRPs frequently occurred through poor communication between primary and secondary care about medication changes [23]. Although many researchers have recognized the impact of communication between clinical pharmacists and physicians on patients’ medication decisions, they have not paid attention to communication details (such as communication time and communication frequency) that affect the final medication decisions and patients’ health outcomes [13, 15].

### Theories for understanding clinical pharmacist-physician communication

Polymedicine patients are typically faced with complex medication decisions and require collaboration between physicians and pharmacists to support decision-making. There is a lack of information on pharmacist-physician communication and the communication factors that affect the use of medicines.

Traditional shared decision-making models are limited to the patient-physician dyad, yet care is increasingly planned and delivered through interprofessional teams [24]. France et al. proposed a model linking multiple professionals for an interprofessional approach to shared decision-making (IP-SDM) in primary care. They argued that such a model could further improve the quality of care by fostering continuity in the decision-making process within the multiple components of the health care system [25]. Six key assumptions underlying the IP-SDM model include 1) Equipoise, which refers to a situation where a decision point with more than one option exists and for which potential benefits and harms should be weighed; 2) Exchange of information about the options relevant to the patient’s health condition; 3) Values clarification by individuals involved in the decision-making process; 4) Feasibility of the options during the decision-making process; 5) Achieving consensus among all of the health care providers. 6) Evaluating the implementation of fidelity and health outcomes [24, 26]. Obviously, IP-SDM can make a difference in the decision-making of polypharmacy in the treatment of patients, which can guide care providers to cooperate intentionally and share knowledge and decision-making.

Furthermore, relational coordination (RC) theory provides us with concrete dimensions to understand the possible influencing factors in the process of cooperation and communication between clinical pharmacists and physicians. Relational coordination is an organizational performance theory used across industries, including health care, that describes the management of interdependence between people and tasks [27, 28]. Cramm et al.’s study indicated that the delivery of chronic illness care was positively correlated with RC [29]. RC has seven dimensions, four of which measure the frequency, timeliness, accuracy, and problem-solving nature of communication. Three dimensions measure the degree of shared goals, shared knowledge, and mutual respectability for assessing the quality of the underlying relationships. These dimensions of communication based on RC theory are suitable for understanding communication between clinical pharmacists and physicians.

To sum up, three research questions were put forward: (1) What is the current mode of communication between clinical pharmacists and physicians? (2) Is communication between clinical pharmacists and physicians effective in reducing the occurrence of DRPs? (3) What are the communication factors between clinical pharmacists and physicians affecting the occurrence of DRPs? This study aimed to use semistructured interviews to explore the current communication mode between clinical pharmacists and physicians based on the IP-SDM model and RC theory and to conduct randomized controlled trial (RCT) to explore their effects on reducing DRPs.

## Methods

Quantitative and qualitative methods were used in this study. A semistructured interview was conducted to determine the current mode of communication between clinical pharmacists and physicians. Guided by the results of the qualitative study, we carried out training for clinical pharmacists based on IP-SDM before the intervention. Then we conducted a single-blind RCT to evaluate the effectiveness of communication intervention between clinical pharmacists and physicians by comparing the occurrence of DRPs during medicine reconciliation. Finally, we used logistic regression analysis to analyze the influencing communication factors of DRPs.

### Semistructured interviews

A semistructured interview with clinical pharmacists was conducted to understand the current model of communication between clinical pharmacists and physicians. Six clinical pharmacists were invited to participate in the interview that was conducted by two interviewers. Interview questions were open-ended, adapted from [30], which focused on the subjective experiences and perceptions of clinical pharmacists, including the shared decision-making mode and frequencies and the communication process between clinical pharmacists and physicians (the interview guideline is available in Appendix 1). To reduce interview bias, we primarily adopted the following methods: (1) Interviewer training: Through training, interviewers were allowed to memorize interview content, improve interview skills, and express interview questions clearly and fully; (2) Purposive Sampling: A full consideration of the title, education and length of service of interviewees was undertaken to ensure that the sample was representative. (3) Process control: Interviews were conducted at respondents’ workplaces to avoid the impact of environmental changes on their subjective perceptions. Different questioning styles were used to verify the answer to the same question. After the interview, the data were fed back to the interviewee for reverification and confirmation. The original data and coding results were fed back to the research team in a timely manner so that they could discuss whether the interview content conformed to the research theme and revise the interview guidelines if necessary.

The thematic qualitative analysis steps are as follows: (1) interviews were transcribed verbatim; (2) core research team members read the transcripts and listened to the audios to familiarize themselves with the interviews; (3) core team members thematically coded the data; (4) the entire team thematically coded a subset of six interviews; (5) the team codes were used to develop a working analytic framework; (6) two team members recoded the data; and (7) finally, the data were stored, organized and presented to the entire team for discussion and refinement using NVIVO 12 Software.

### RCT

#### Study settings

A randomized controlled trial was conducted between April 2020 and December 2020 at a tertiary teaching hospital in Xinjiang, China. The hospital has 1500 beds and 2000 open beds. In 2017, there were 64,800 discharged patients and 907,200 outpatient and emergency patients. This trial was approved by the ethics committee of the surveyed Hospital (kj2020–087-03) and registered in the China clinical trial registry (www.chictr.org.cn, number ChiCTR2000035321 date: 2020/08/08). Written informed consent were obtained from all subjects.

#### Participants

**Fig. 1:**
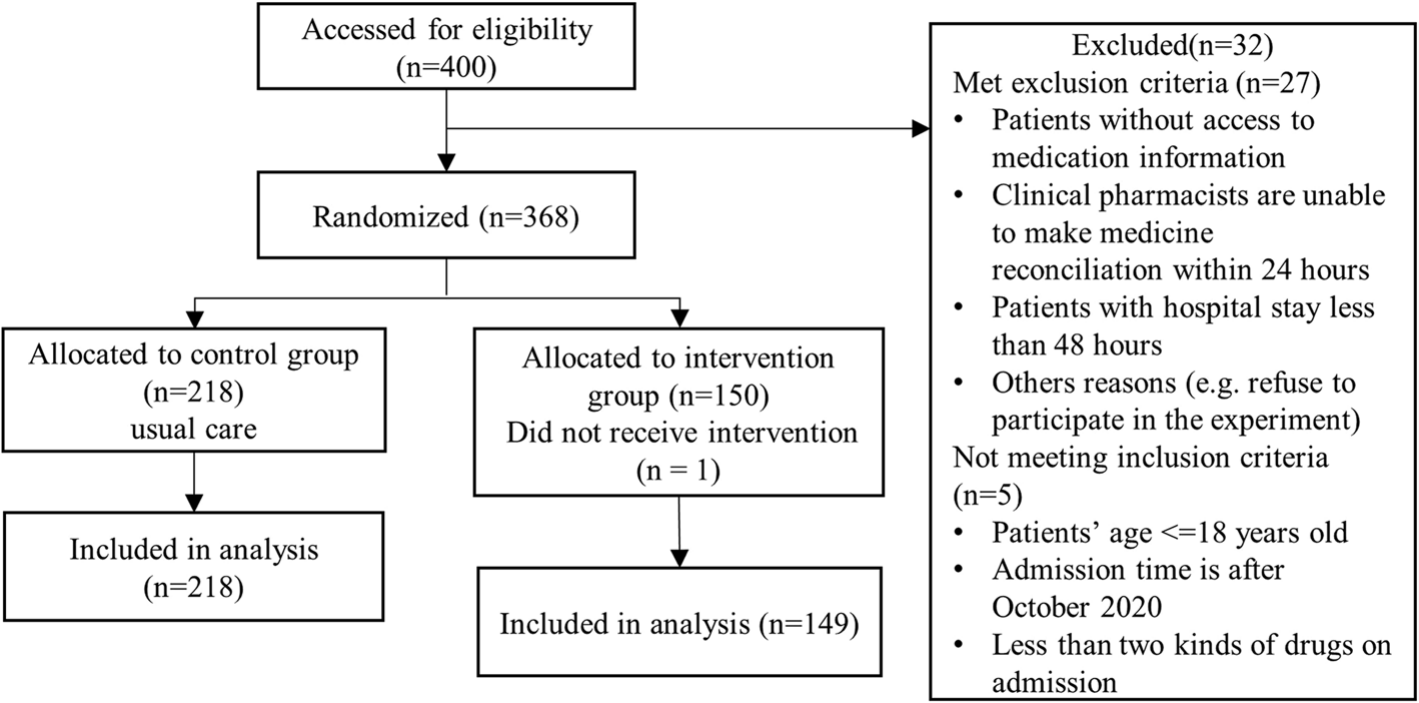
Study flow diagram

Considering the limited number of clinical pharmacists, we designed this unequal randomization rate for reasons of labor cost and benefit of the intervention. We used PASS to calculate the recommended sample size of 375 in the intervention group and the control group according to the ratio of 1:1.5. Considering the loss of follow-up, we recruited 400 patients. Patients were recruited from chronic disease inpatient units (e.g., cardiology department, nephrology department, endocrine department, etc.) between April 2020 and December 2020. Patients were assessed for eligibility as shown in the study flow in Fig. 1, and prescription information for medication reconstitution was divided into two main categories (including the participant’s preadmission medication history list and physician’s prescriptions postadmission). Finally, 368 patients were enrolled and randomly assigned. One patient withdrew, leaving 367 patients for the intention of the study analysis.

#### Blinding

The research team informed the clinical pharmacists and physicians about the grouping of each patient in the communication stage. Clinical pharmacists collected and compared the histories of patients’ medication lists and the list of physicians’ prescriptions on the spot, sorted out and generated a list of patients’ DRPs, and communicated with medical staff on unintentional medication differences.

#### Intervention measures

##### Intervention group

**Fig. 2:**
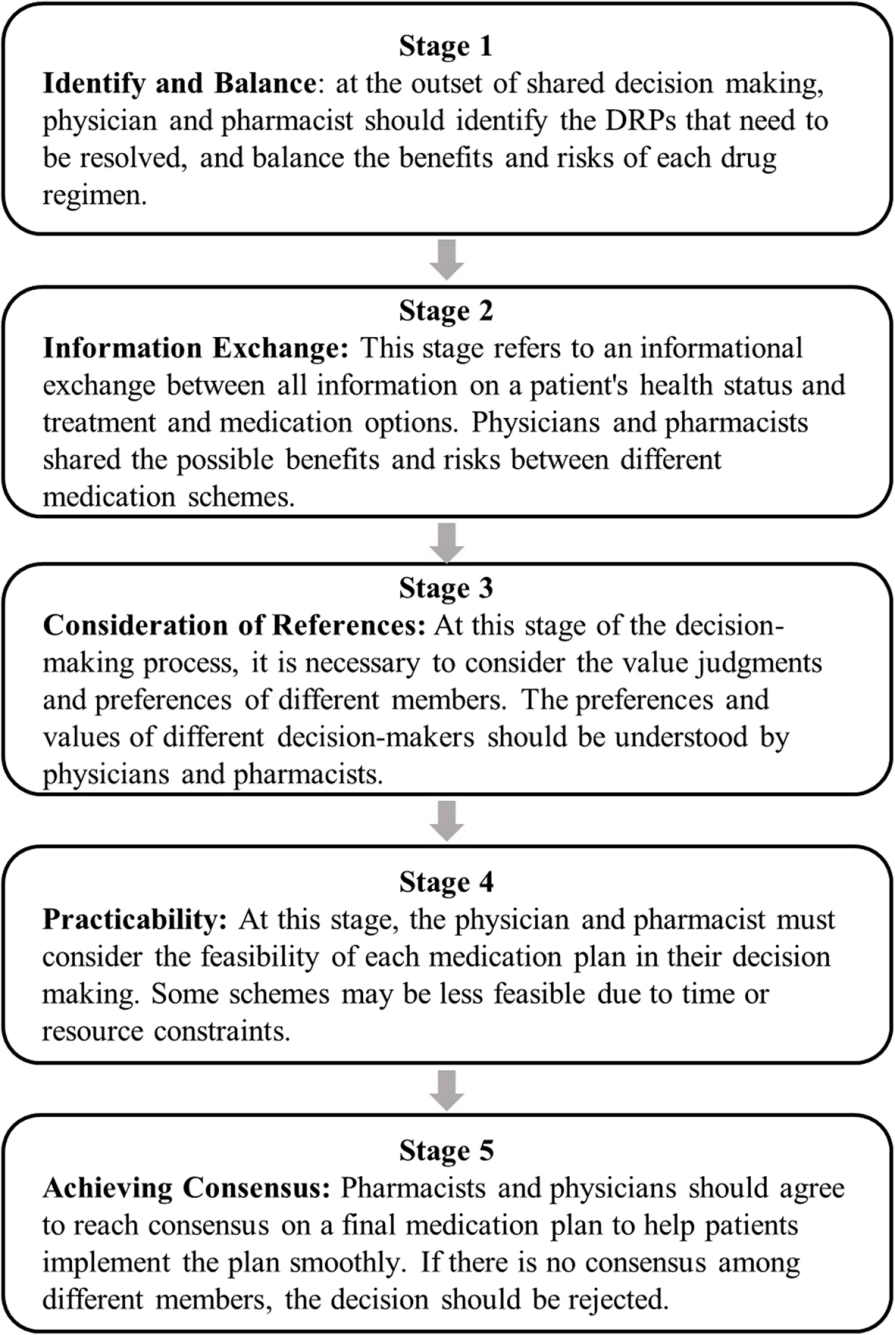
Five stages of IP-SDM

Before the intervention, a three-hour workshop on shared decision-making was delivered in the pharmacy department, training five stages of communication based on IP-SDM (Fig. 2). Then qualified clinical pharmacists conducted the following interventions: (1) Obtained the basic patient information such as patient name, gender, age, bed number, admission diagnosis, allergy history, etc. through the hospital electronic medical record system; (2) Interviewed patients to create a preadmission medication list, which includes drug name, strength, single dose, frequency, dosage form, route of administration, course of treatment, and basis/source of the drug; (3) Based on professional knowledge, clinical pharmacists referred to pharmacy databases (such as rational drug use systems and medication assistants), Chinese Pharmacopoeia and relevant drug inserts to evaluate the drugs in the list; (4) Compared differences in medication use preadmission and postadmission, and assessed and recorded the DRPs; (5) recommend the DRPs to physicians for immediate implementation; discuss the potential solutions of DRPs with physicians; and finally reach consensus on a medication decision and help patients implement the plan smoothly.

##### Control group

The patients received a usual pharmaceutical care provided by physicians and nurses. Nurses collected basic patient information (name, gender, age, bed number, admission diagnosis, allergy history, etc.) and established medication lists of preadmission and postadmission, and physicians assessed and recorded DRPs based on the taxonomy guidelines.

### Data collection

#### Medication information of patients

Preadmission medication history was collected for all eligible patients within 24 hours of admission, which was completed by clinical pharmacists. The list included patients’ personal information, medication history and assessments of DRPs. Furthermore, for the intervention group, clinical pharmacists collected another medication list of postadmission and evaluated the DRPs. The medication information collection list is based on [31], which enables accurate and complete recording of medication-related information.

#### Outcomes of intervention

Clinical pharmacists recorded the primary outcome measures of DRPs by comparing the patient medication list from the hospital information system. Researchers investigated the unplanned readmission of patients within 30 days after patients were discharged from the hospital through telephone follow-up. Secondary outcomes were the level of communicative factors, including communication ways, physicians’ feedback, consensus of communication, communication contents and communication time. Since the communication content included four kinds of items (raising medication differences or medication-related issues with the physician; providing clinicians with evidence and information about medication differences and problems; discussing patient medication preferences; weighing different drug use decisions with physicians) and clinical pharmacists could choose one or more items, we set four levels to define this variable (1–4 kinds). Measurement items for these categorical variables were adapted from research [32], with a score of 0–1 for binary variables, and a score of 1–3 for tertiary variables. The above data were collected from the questionnaire that was available in Appendix 2.

### Statistical analysis

Data were analyzed using R-4.0.0, and base data were presented as the mean or as percentages within groups. Student’s sample t test was used to evaluate the differences between continuous variables. Fisher’s exact and chi-square tests were used to compare categorical data, and a *p*-value < 0.05 was considered to be statistically significant. A binary logistic regression analysis was performed to examine the influencing factors of communication between pharmacists and physicians.

## Results

### The mode of communication between clinical pharmacists and physicians

We mainly focused on pharmacists’ views on the way and frequency of shared decision-making with physicians and the handling of disputes when dealing with DRPs. All the pharmacists expressed that they frequently communicated the DRPs with physicians in the medical wards. However, if the physicians were busy or unavailable in the medical wards, they would choose other ways (such as phone calls or WeChat) to connect with physicians later. Some pharmacists have suggested advanced software developments in the health information system to contact physicians, which could help solve the dilemma of daily attendance in the medical ward and the distance between the pharmacy department and the medical department. Regarding the communication frequency with physicians, pharmacists expressed that they had no regular communication frequency with physicians. If there is the need to discuss DRPs or the uncertainties of how to deal with DRPs, pharmacists will keep in touch with the physicians. Despite this, they all said they could not communicate fully and effectively in most cases.

The communication mode led by pharmacists between clinical pharmacists and physicians is selectively based on different DRP types. If the DRPs were an easily identified problems, such as the repetition of medicine use or the wrong frequency or way of medicine use, which are clearly defined in the instructions of medicines, the pharmacists would deal with patients directly and provide relevant medical education and proper medical suggestions for patients and then relay the information to physicians. Regarding the problems that were not easy to identify, the pharmacists chose to discuss the DRPs with physicians. However, it is worth noting that most pharmacists tried to hide the potential DRPs before they discussed them with physicians. When disagreement arises about DRPs, both pharmacists and physicians would be required to provide evidence of their respective opinions The benefits and disadvantages of each option would be compared to decide which option should be adopted. One pharmacist said that if the DRPs would not affect the overall treatment plan, he would follow the physicians’ recommendations. However, if the DRP is one serious problem that would cause potential harm for the patient, he would insist on his opinion and reach the consensus based on the high evidence-based medical support.

### The effect of communication intervention on DRPs

The demographic characteristics of the intervention and control groups were mainly 57.5% male, 70.1% high school education, nearly 80% medical insurance for urban employees, and 58.5 years old. There were no significant differences in baseline characteristics between the intervention and control groups (*p* > 0.05) (Table 1). Among 367 participants, 44 patients had DRPs. RCT found that there was a significant decrease in the incidence of DRPs in the intervention group compared to the control group (15.6% vs. 0.07%, *p* = 0.02). However, there were no statistically significant differences in unplanned readmission (*p* > 0.05) (Table 2).

**Table 1:** Basic characteristics of patients (*n* = 367)

Variables	Control group (***n*** = 218)	Intervention group (***n*** = 149)	Total	***p***
**Gender (%)**				0.173
Male	119 (54.6)	92 (61.7)	211 (57.5)	
Female	99 (45.4)	57 (38.3)	156 (42.5)	
**Age**	58.00 [51.25, 70.50]	59.00 [52.00, 71.00]		0.597
**Medical Insurance (%)**				0.539
Medical insurance for Urban employees	121 (77.6)	14 (73.7)	135 (77.1)	
Medical insurance for urban and rural residents	9 (5.8)	2 (10.5)	11 (6.3)	
The new rural cooperative medical insurance	3 (1.9)	1 (5.3)	4 (2.3)	
Self-paying	14 (9.0)	1 (5.3)	15 (8.6)	
Non-local medical insurance	4 (2.6)	0 (0.0)	4 (2.3)	
Individual medical insurance	5 (3.2)	1 (5.3)	6 (3.4)	
**Education (%)**				0.236
College or above	9 (4.2)	10 (6.8)	19 (5.2)	
Some college	39 (18.1)	24 (16.2)	63 (17.3)	
High school or below	154 (71.3)	101 (68.2)	255 (70.1)	
Illiteracy	14 (6.5)	13 (8.8)	27 (7.4)	
**Occupation(%)**				0.567
Government	36 (16.7)	21 (14.3)	57 (15.7)	
Professional and technical personnel	26 (12.0)	28 (19.0)	54 (14.9)	
Service industry personnel	19 (8.8)	13 (8.8)	32 (8.8)	
Agriculture	89 (41.2)	59 (40.1)	148 (40.8)	
Production and transportation	18 (8.3)	12 (8.2)	30 (8.3)	
Other	28 (13.0)	14 (9.5)	42 (11.6)

**Table 2:** The effect of communication intervention on primary outcomes (*n* = 367)

Primary Outcomes	Level	Control Group	Intervention Group	X^2^	*P*
n		218	149		
Drug Related Problems	Yes	34	10	5.81	0.02
	No	184	139		
Unplanned Readmission within 30 Days	Yes	7	8		
	No	211	141	0.57	0.45

In the intervention group, the communication ways of clinical pharmacists and physicians were mostly face-to-face (91.3%), and physicians always provided feedback (98.6%). In most cases (97.2%), the two sides can reach a consensus on the solution of DRPs. In addition, there were two (33.8%) or fewer (35.1%) kinds of communicational content between clinical pharmacists and physicians, and the communication time was usually approximately 5.8 minutes. Univariate analyses showed that communication time and age were significantly correlated with DRPs (*p* < 0.001) (Table 3). Therefore, we included these two variables in the regression model. Logistic regression analysis showed that communication time (OR = 13.22, *p* < 0.001) between clinical pharmacists and physicians was the main factor influencing the incidence of DRPs in the intervention group. However, the significance of age disappeared (Table 4).

**Table 3:** The univariate analysis of the intervention group on DRPs (*n* = 149)

Variables	N (mean-%)	*p*
*Demographic variables*		
Age	59	0.03*
Gender		0.65
*Male*	92 (61.7)	
*Female*	57 (38.3)	
Medical Insurance		0.3
*Medical insurance for urban employees*	14 (73.7)	
*Medical insurance for urban and rural residents*	2 (10.5)	
*The new rural cooperative medical insurance*	1 (5.3)	
*Self-paying*	1 (5.3)	
*Non-local medical insurance*	0 (0.0)	
*Individual medical insurance*	1 (5.3)	
Education		0.34
*College or above*	10 (6.8)	
*Some college*	24 (16.2)	
*High school or below*	101 (68.2)	
*Illiteracy*	13 (8.8)	
Occupation		0.64
*Government*	21 (14.3)	
*Professional and technical personnel*	28 (19.0)	
*Service industry personnel*	13 (8.8)	
*Agriculture*	59 (40.1)	
*Production and transportation*	12 (8.2)	
*Other*	14 (9.5)	
*Communication variables*		
Communication Ways		0.56
*Face to Face*	136 (91.3)	
*Phone/WeChat*	13 (8.7)	
Physicians’ Feedback		1
*Yes*	143 (96)	
*No*	6 (4)	
Consensus of Communication		0.38
*Yes*	141 (94.6)	
*No*	8 (5.4)	
Communication Contents		0.77
*One Kind*	53 (35.6)	
*Two Kinds*	50 (33.6)	
*Three Kinds*	34 (22.8)	
*Four Kinds*	12 (8)	
Communication Time (minutes)	5.80 (3.35)	< 0.001*

**Table 4:** Logistic analysis of the influence of communication intervention on DRPs (*n* = 149)

Variables	B	Wald	OR (95% CI)	*P*
Communication time	−0.95	20.78	0.39 (0.25, 0.56)	< 0.001
Age	−0.05	2.23	0.95 (0.88, 1.01)	0.13

## Discussion

This study found that the current mode of communication between clinical pharmacists and doctors is face-to-face led by clinical pharmacists. Furthermore, we confirmed that communication between clinical pharmacists and physicians in medication decision-making can reduce DRP incidence, and that length of communication time is a major factor. The longer the duration of the communication, the fewer DRPs are likely to occur.

### Problems with current communication modes

Qualitative research results found that the current mode of communication between clinical pharmacists and doctors is mainly selective face-to-face communication. Previous studies also highlighted the positive significance of interdisciplinary medical team collaborations led by clinical pharmacists [33, 34]. However, we found that the current mode between clinical pharmacists and physicians still has many problems. Many pharmacists said that they could not fully communicate with physicians. We consider the possible reasons: First, clinical pharmacists’ participation in MR is still in its infancy in developing countries. Due to different tasks, the interaction between clinical pharmacists and physicians on drug use decisions is relatively random and nonstandard. In addition, most clinical pharmacists have not received professional training and have failed to communicate effectively with physicians. This can result in the exclusion of pertinent information on DRPs. Another study suggests that interpersonal relationships (such as trust) between clinical pharmacists and physicians are also an essential factor, and physicians will be more inclined to communicate and cooperate with familiar pharmacists [35]. The application of the IP-SDM model in this study shows promising results. This model provides a standard guide for clinical pharmacists and physicians in dealing with DRPs and improves the efficiency of communication.

### Communication between clinical pharmacists and physicians can reduce the occurrence of DRPs

Although many researchers have conducted extensive research on communication between pharmacists and patients [36], our study suggested that communication between medical service providers was also critical for providing medical services, and there were factors affecting communication between clinical pharmacists and physicians. The RCT results indicated that communication between clinical pharmacists and physicians in medication decision-making could reduce the incidence of DRPs. Previous studies suggested that pharmacists’ participation could reduce the incidence of medication errors [4], and our study further confirmed the positive effect of improving the communication between clinical pharmacists and physicians. The occurrence of DRPs is mainly due to physicians’ inadequate knowledge of medication information or pharmacy-related knowledge. Clinical pharmacists can help physicians issue appropriate prescriptions. No significant change was observed in the other primary outcomes, 30-day unplanned readmission rates after the intervention, which was similar to the results of previous studies on MR. This may reflect a gap between reducing DRPs and the clinical outcomes of patients. A 2019 overview showed that MR failed to achieve a clear improvement in health outcomes [37]. Hawes’s research reported that the intervention group showed a nonsignificant reduction in health service utilization [38]. Additionally, Gillespie et al. suggested that the time scale of the follow-up period (30-day mark) is too short in the usual study [39]. However, in any case, the reduction of DRPs will have a more favorable impact on the health of polymedicine patients.

### Communication time is a key factor

Based on the dimensions that measure communication effectiveness by RC, the logistic regression results indicated that the length of communication time significantly negatively affected the occurrence of DRPs. This suggests that the longer the communication time is, the fewer DRPs that occur, indicating the importance of full communication between clinical pharmacists and physicians to reduce drug disparities. Aburuz et al. noted in their research that pharmacists’ recommendations often lead to lower actual implementation rates due to delays in communicating DRPs between health care providers, and our results are consistent with these findings [40]. Due to the different professional backgrounds, inconsistent knowledge of patient information, and some objective factors (such as the geographical location of the office, the business scope of doctors and pharmacists), the communication between them is not sufficient [41]. Furthermore, we speculate that lengthening the communication time may help pharmacists feedback some potential DRPs. Previous interview results indicated that pharmacists might be hiding potential DRPs, which may be due to limited communication time.

### Implications

While there is a significant role for collaboration between clinical pharmacists and physicians in IP-SDM, few studies focus on the specific details of the communication process between clinical pharmacists and physicians and the related communication factors that affect outcome variables (DRPs). The results of this study provide the following insights: First, we should pay attention to the influencing factors in the process of communication between clinical pharmacists and physicians, since improving the effectiveness of communication will help to improve and facilitate the outcome of MR. Second, to ensure that there is sufficient communication between clinical pharmacists and physicians, we suggest two aspects of team building and information technology support: 1) hospitals should establish a professional team including clinical pharmacists and physicians for the medication decision-making of patients with multiple drugs, conduct standardized training for professionals, clarify the division of responsibilities, and improve the work efficiency of professionals. A study reported that pharmacists completing MR had reduced physician visits and increased clinical time for other health team members [42]. 2) Establish an information-sharing electronic platform based on the interaction of patient medication information records and decision-making to realize the real-time sharing of MR information between clinical pharmacists and physicians and timely communication in order to reach a consensus.

### Strengths & Limitations

Previous studies paid more attention to the changes in clinical outcomes by pharmacist-led MR and emphasized the important role of clinical pharmacists [19]. However, few studies focus on the communication details of the collaboration between clinical pharmacists and physicians during the process of pharmaceutical care. To our knowledge, this is the first RCT study to explore the influence of communication between clinical pharmacists and physicians on DRPs based on the IP-SDM model and RC theory in China. Our research results will provide a reference in theory and practice to improve the collaboration between clinical pharmacists and physicians and improve the efficiency and value of MR.

There are several limitations in our study. First, this study is a single-center randomized controlled trial, and further multicenter randomized controlled trials are needed to verify the universality of the experimental results. In addition to the planned 30-day readmissions, we can also consider other clinical outcomes that may be affected by communication between clinical pharmacists and physicians. Third, although this study found that communication time may be a key factor, extending communication time is not necessarily the best way. Future studies should consider other factors that can improve the inadequate communication between clinical pharmacists and physicians.

## Conclusion

Based on the IP-SDM model and RC theory, this study investigated the current mode and influencing communication factors between clinical pharmacists and physicians in MR cooperation in China and tested its communicative effect through RCT. This research provides the modes guidance and evidence support for the communication between clinical pharmacists and physicians, and provides theoretical basis and practical enlightenment for the development of pharmaceutical services.

## Supplementary Information

**Additional file 1.** 

**Additional file 2.** 

**Additional file 3.** 

## Data Availability

All data generated or analyzed during this study are included in this published article and its supplementary information files.

## Abbreviations

DRPs: Drug-related problems
RCT: Randomized controlled trial
MR: Medication reconciliation
IP-SDM: Interprofessional approach to shared decision making
RC: Relational Coordination
