# Supplementary material for: The impact of clinical pharmacist-physician communication on reducing drug-related problems: a mixed study design in a tertiary teaching Hospital in Xinjiang, China
Source: BMC Health Serv Res. 2022 Sep 14;22:1157. doi: 10.1186/s12913-022-08505-1 (PMC9472438; doi:10.1186/s12913-022-08505-1)
Supplement: Supplementary file 1 — Additional file 1. [file 12913_2022_8505_MOESM1_ESM.docx]

Appendix 1: Guidelines of Semi-structured Interview

In order to understand the current model of communication between clinical pharmacists and physicians we conducted a semi-structured interview with clinical pharmacists. The guidelines are as follows：

1. If there are problems related to drugs, what would you do first? When or under what circumstances would you choose to contact a physician?
2. How do you contact the clinician?
3. What do you think is the most convenient way to contact? Do you think the current contact information needs to be improved?
4. How often do you contact a physician? Is it through the next ward face-to-face contact? Telephone? WeChat? When will you choose face to face? When will you choose phone or WeChat?
5. How do you know that the physicians may need to communicate with you about the drug related problems of patients?
6. When your opinion is inconsistent with the physicians, and how to solve the problem?
