# Supplementary material for: The impact of clinical pharmacist-physician communication on reducing drug-related problems: a mixed study design in a tertiary teaching Hospital in Xinjiang, China
Source: BMC Health Serv Res. 2022 Sep 14;22:1157. doi: 10.1186/s12913-022-08505-1 (PMC9472438; doi:10.1186/s12913-022-08505-1)
Supplement: Supplementary file 2 — Additional file 2. [file 12913_2022_8505_MOESM2_ESM.docx]

**Appendix 2**

**Patients**

□ intervention group

□ control group

**Demographic information**

1. Patients ID：
2. Clinical departments：1. Oncology 2. Endocrinology 3. Neurology 4. Cardiology NO.1 5. general 6. Nephrology 7. Cardiology NO.3 8. Cardiology NO.2
3. Gender: 1. Male 2. Female
4. Inpatient number：
5. Age：
6. Date of admission： Mm / DD / 2020
7. Medical Insurance：1. medical insurance for urban employees 2. medical insurance for urban and rural residents 3. The new rural cooperative medical insurance 4. self-paying 5. non-local medical insurance 6. Individual medical insurance
8. Admission condition: 1. Critical 2. Severe 3. Urgent 4. General
9. Whether there are allergic drugs or food: 1. No 2. Yes
10. Education: 1. College or above 2. Some college 3. High school or below 4. Illiteracy
11. Occupation:1. Government 2. Professional and technical personnel 3. Service industry personnel 4. Agriculture 5. Production and transportation 6. Other
12. Have you lived in a hospital in the past three years: 1. No 2. Yes
13. Unplanned readmission: 1. No 2. Yes (notes: for the pre group patients who are dry 30 days after discharge)

**Medication information**

1. Medication information before admission

| Drug code | Drug name | Dosage | Frequency | Route of administration | Course of treatment | Drug-related problems? |
| --- | --- | --- | --- | --- | --- | --- |
| 1 |  |  |  |  |  |  |
| 2 |  |  |  |  |  |  |
| … |  |  |  |  |  |  |

1. Medication information after admission

| Drug code | Drug name | Dosage | Frequency | Route of administration | Medication difference？ | Drug-related problems? |
| --- | --- | --- | --- | --- | --- | --- |
| 1 |  |  |  |  |  |  |
| 2 |  |  |  |  |  |  |
| … |  |  |  |  |  |  |

**Clinical pharmacist**

1. Forms of Communication：1. Face to Face 2. Phone/WeChat

2. Feedback from Physicians: 1. No 2. Yes

3. Achieve a Consensus: 1. No 2. Yes

4. Communication Time(minutes):

5. Contents of Communication (multiple choice):

-  Raise medication differences or medication related issues with the physician；
-  Provide clinicians with evidence and information about medication differences and problems；
-  Discuss patient's medication preferences (e.g. reimbursement, medication habits) ；
-  Weighing different drug use decisions with physicians；

6. Adjustments in The Prescription System（multiple choice）:

-  Deletion / addition / replacement of drugs；
-  Drug monitoring and reporting；
-  Adjust dosage form / dose / route of administration；
-  Maintain the original state
